# Declining age-adjusted mortality but persistent inequalities in chronic obstructive pulmonary disease in the United States, 1999–2024: a CDC WONDER analysis contextualized by GBD 2023

**DOI:** 10.3389/fpubh.2026.1852919

**Published:** 2026-06-18

**Authors:** Hongnan Zhang, Fan Xiao, Lieliang Zhang, Hong Zhu

**Affiliations:** 1 Department of Anesthesiology, The Second Affiliated Hospital of Nanchang University, Nanchang, China; 2 The Second Affiliated Hospital of Nanchang University, Nanchang, China; 3 Nanchang University, Nanchang, China; 4 Department of Neurosurgery, The Second Affiliated Hospital of Nanchang University, Nanchang, China

**Keywords:** CDC WONDER, chronic obstructive pulmonary disease, Global Burden of Disease, health inequality, Joinpoint regression, mortality, rural health, sex differences

## Abstract

**Background:**

Chronic obstructive pulmonary disease (COPD) remains a leading cause of premature mortality and disability worldwide. National averages in the United States often obscure heterogeneity across sex, geography, rurality, and age. We examined U.S. COPD mortality trends from 1999 to 2024 and contextualized findings using Global Burden of Disease (GBD) 2023 data.

**Methods:**

We conducted a serial cross-sectional ecological study using Centers for Disease Control and Prevention Wide-ranging Online Data for Epidemiologic Research (CDC WONDER) mortality data and investigator-generated GBD 2023 visual outputs. Age-adjusted mortality rates (AAMRs, per 100,000 population, standardized to the year 2000 U.S. standard population) were assessed by sex, age group, race and ethnicity, census region, state, and urbanization. Joinpoint regression (National Cancer Institute Joinpoint software, v5.2.0) was used to estimate annual percent changes (APCs), average annual percent changes (AAPCs), and 95% confidence intervals (CIs). GBD 2023 figures contextualized global prevalence, socio-demographic development and COPD disability-adjusted life-years (DALYs), and regional risk-factor contributions.

**Results:**

From 1999 to 2024, COPD deaths rose from 99,550 to 132,115 (+32.71%), while the AAMR declined from 56.38 to 45.26 per 100,000 (AAPC, −0.88; 95% CI, −1.15 to −0.61). Men showed greater improvement (74.09 to 47.97; AAPC, −1.59) than women (46.03 to 43.17; AAPC, −0.31), whose rates rose until 2016 before declining. Urban–rural disparities widened: metropolitan areas improved (54.91 to 47.16; AAPC, −0.61), whereas nonmetropolitan areas worsened (62.73 to 72.41; AAPC, +0.77). In 2024, state-level AAMRs ranged from 18.51 to 85.39 per 100,000. Age gradients were steep, from 0.08 among adults aged 25–34 years to 523.69 per 100,000 among those aged ≥85 years. GBD 2023 data confirmed a non-linear pattern across socio-demographic development, with burden driven by smoking, ambient particulate pollution, occupational exposures, and household air pollution.

**Conclusion:**

U.S. COPD mortality has improved nationally, yet progress remains uneven. Population ageing, slower decline among women, and persistent rural and geographic inequalities sustain the burden. Public health strategies should prioritize women-centered case finding, rural access to smoking cessation and pulmonary rehabilitation, and place-based exposure reduction.

## 1 Introduction

Chronic obstructive pulmonary disease (COPD; International Classification of Diseases, Tenth Revision [ICD-10] codes J40 [bronchitis, not specified as acute or chronic], J41 [simple and mucopurulent chronic bronchitis], J42 [unspecified chronic bronchitis], J43 [emphysema], and J44 [other COPD]) remains one of the leading causes of chronic respiratory morbidity, disability, and premature mortality worldwide (1, 2). The GBD 2023 study estimated that chronic respiratory diseases accounted for approximately 4 million deaths globally in 2023, with COPD contributing the largest share. Although therapeutic and preventive strategies have advanced, the global burden of COPD remains substantial, particularly in ageing populations and in settings with persistent tobacco, environmental, or occupational exposures (1–7). COPD is also increasingly understood as a heterogeneous condition shaped by life-course influences rather than by smoking alone (1–7).

In the United States, prior work has shown that COPD mortality is not distributed evenly. Important differences have been documented across sex, race and ethnicity, census region, state, and rural–urban residence (8–15). Those disparities are not trivial background variation. They suggest that the national average may conceal meaningful gaps in prevention, diagnosis, access to care, and exposure control (8–15).

The sex pattern in COPD deserves particular attention. Although men have historically carried a higher mortality burden, a large body of literature has shown that women may differ in exposure history, susceptibility, symptom profile, diagnostic pathway, and disease trajectory (16–22). Women are also more vulnerable to under-recognition when COPD does not match older clinical stereotypes, which may delay diagnosis and treatment (17–22). Women remain vulnerable to under-recognition—defined as delayed diagnosis or misattribution of respiratory symptoms—in part because historical clinical stereotypes have framed COPD as a disease of older male smokers. This mismatch between clinical expectation and epidemiologic reality may contribute to later presentation, lower rates of spirometry, and reduced access to guideline-based care among women (22).

Understanding whether mortality trends differ by sex, geography, and urbanization is essential for targeting interventions where they are most needed. Non-smoking-related COPD, including disease linked to air pollution, occupational exposures, biomass smoke, and secondhand smoke, now occupies a more prominent place in the literature (23–32). In parallel, underdiagnosis remains common, and access to spirometry and pulmonary rehabilitation is still uneven, particularly outside major metropolitan areas (33–40). Placing U.S. trends within the Global Burden of Disease (GBD) 2023 framework is important because it allows us to compare the U.S. experience with global age-sex patterns, assess whether socioeconomic development alone explains burden reduction, and identify the relative contributions of smoking versus non-smoking risk factors—insights that inform whether U.S. disparities reflect unique policy gaps or broader structural determinants of COPD.

Placing U.S. trends within the Global Burden of Disease (GBD) 2023 framework provides essential context for interpreting domestic patterns. First, COPD burden worldwide is fundamentally age-structured: prevalence and mortality rise steeply with age, and population ageing can sustain or even increase absolute deaths despite declining age-standardized rates (4, 7). This global phenomenon helps explain why U.S. deaths continue to rise even as age-adjusted mortality falls. Second, the relationship between socioeconomic development and COPD burden is non-linear across countries; high national income does not automatically eliminate disease burden (4, 7). This pattern is relevant to understanding persistent rural and state-level disparities within the United States despite overall high socio-demographic development. Third, smoking remains the dominant risk factor globally, but non-smoking exposures—including ambient particulate matter pollution, occupational particulates and gases, and household air pollution—contribute substantially to COPD burden in many settings (23–32). Recognizing this broader exposure framework is critical for interpreting U.S. geographic inequality, particularly in rural areas and states where tobacco control alone may be insufficient.

The COVID-19 pandemic also warrants consideration in interpreting recent COPD mortality trends. Individuals with COPD were at elevated risk for severe COVID-19 outcomes, and pandemic-related disruptions in healthcare access, delayed diagnosis, and reduced pulmonary rehabilitation utilization may have affected COPD management and outcomes during 2020–2024 (41–43). Additionally, COVID-19 may have been misclassified as the underlying cause of death in some cases where COPD was a contributing condition, potentially affecting the accuracy of COPD mortality estimates during the pandemic period (41–43).

Against this background, we used CDC WONDER mortality data to examine COPD mortality in the United States from 1999 to 2024, with stratification by sex, age, race and ethnicity, census region, state, and urbanization. We then used GBD 2023 outputs to place the U.S. findings in a broader epidemiologic context. Our aim was not simply to describe whether mortality fell, but to ask whether recent improvement was shared equitably across populations and places.

## 2 Materials and methods

### 2.1 Study design, data source and case definition

Mortality data were extracted from the CDC WONDER Underlying Cause of Death database (not the Multiple Cause-of-Death file), using chronic lower respiratory diseases (CLRD) as the underlying cause, identified by ICD-10 codes J40–J44 listed as the underlying cause of death only.

Data were extracted from the CDC WONDER Underlying Cause of Death, 1999–2024 database (provisional release, accessed October 2025). For years 1999–2020, final mortality data files were used; for years 2021–2024, provisional mortality files were used, as final data for these years were not yet available at the time of extraction. ICD-10 codes used: J40 (Bronchitis, not specified as acute or chronic); J41 (Simple and mucopurulent chronic bronchitis); J42 (Unspecified chronic bronchitis); J43 (Emphysema); J44 (Other chronic obstructive pulmonary disease). J45 (Asthma), J46 (Status asthmaticus), and J47 (Bronchiectasis) were excluded. To ensure conceptual alignment with the GOLD COPD case definition and with prior age-adjusted COPD mortality literature, we applied three *a priori* restrictions to our CDC WONDER query: (1) decedents aged ≥25 years; (2) ICD-10 codes J40–J44 as the underlying cause only (excluding J45–J47 — asthma and bronchiectasis); and (3) the Underlying Cause-of-Death file only (excluding any-mention/Multiple Cause-of-Death queries). These three restrictions, while standard in the COPD mortality literature, yield total death counts that are systematically lower than American Lung Association (ALA) tabulations using broader CLRD definitions, all-ages denominators, or multiple-cause queries. Readers comparing our absolute death counts with ALA’s published figures should account for these definitional differences.

Global Burden of Disease 2023 estimates were referenced for contextual comparison (IHME Results Tool, extracted April 3, 2026).

This was a serial cross-sectional ecological study based on publicly available, deidentified aggregate data. U.S. mortality data were obtained from CDC WONDER and covered calendar years 1999 through 2024. COPD deaths were defined using ICD-10 codes J40–J44 as the underlying cause of death, consistent with prior U.S. surveillance work (8, 12).

**Figure 1:**
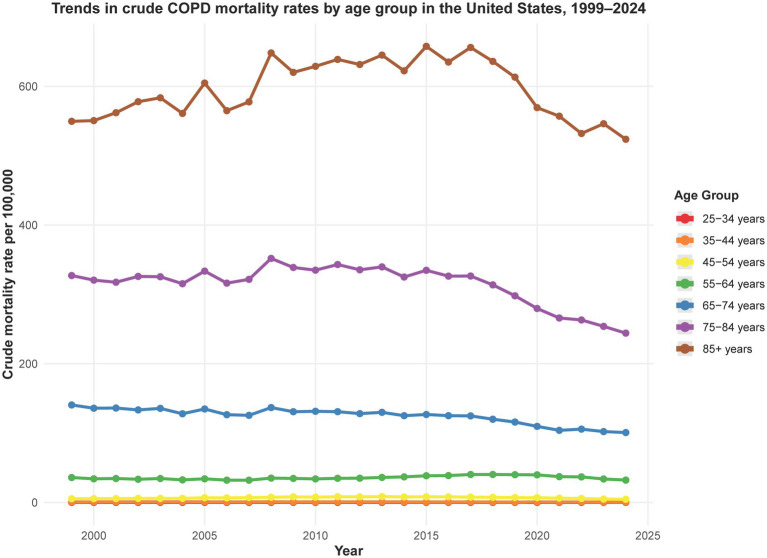
Age-specific COPD mortality trends in the United States, 1999–2024. Crude mortality rates per 100,000 population are shown for adults aged 25–34, 35–44, 45–54, 55–64, 65–74, 75–84, and 85 + years. Joinpoint-derived APCs and 95% CIs describe temporal changes within each age group.

**Figure 2:**
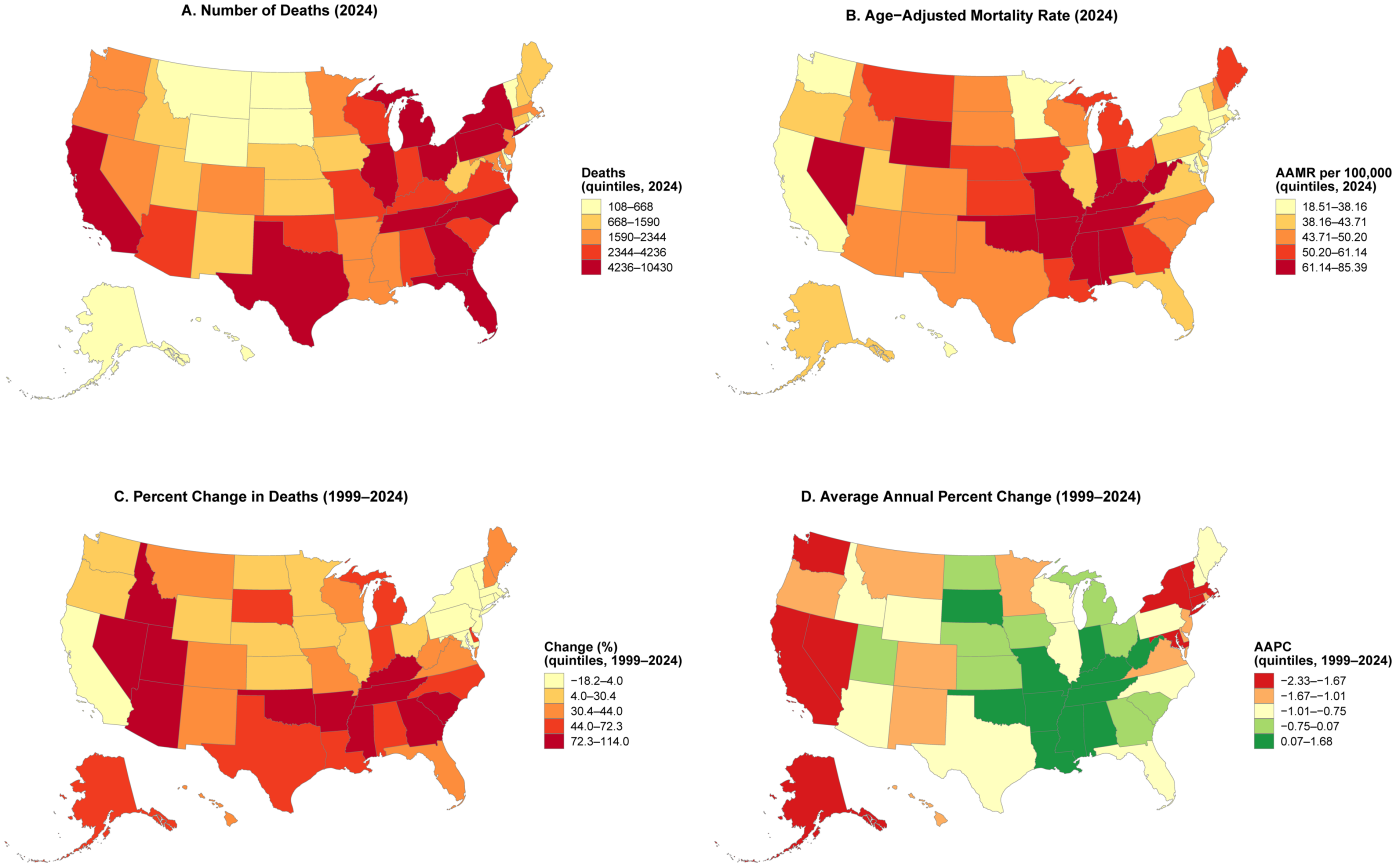
State-level geographic variation in COPD mortality in the United States, 1999–2024. **(A)** Number of COPD deaths in 2024. **(B)** Age-adjusted mortality rate per 100,000 population in 2024. **(C)** Percent change in deaths between 1999 and 2024. **(D)** Average annual percent change in mortality from 1999 to 2024.

**Figure 3:**
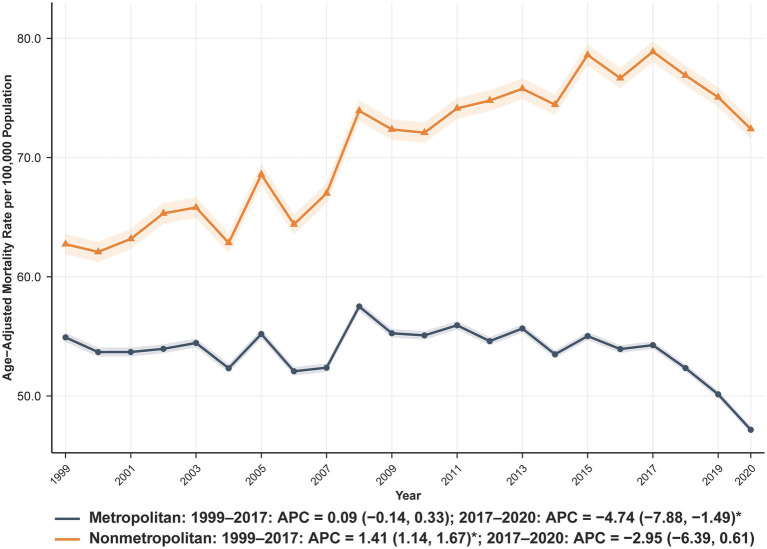
Urban–rural differences in COPD mortality trends in the United States, 1999–2020. Joinpoint trends are shown separately for metropolitan and nonmetropolitan populations. Trend visualization was available through 2020; segment-specific APCs should therefore be interpreted within that interval.

**Figure 4:**
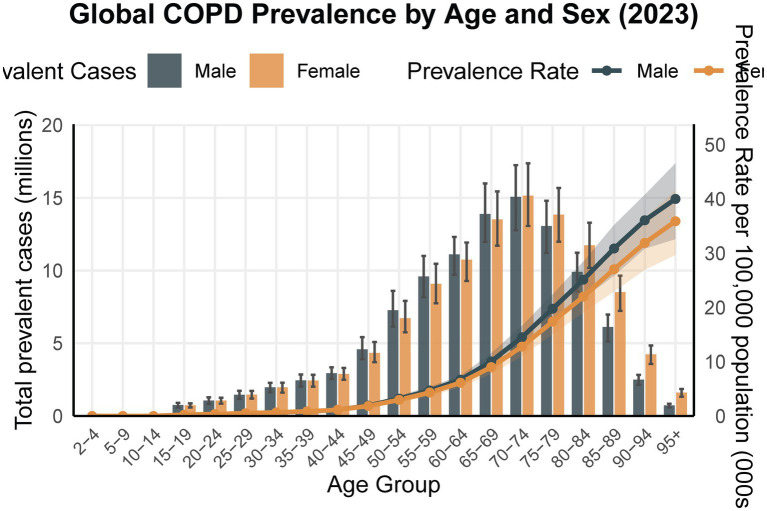
Global COPD prevalence by age and sex, 2023.

GBD 2023 data were accessed via the Institute for Health Metrics and Evaluation (IHME) GBD Results Tool.^1^ These outputs were used solely for contextual interpretation to place U.S. mortality patterns within a broader global epidemiologic framework. GBD estimates were not statistically pooled or directly compared with CDC WONDER mortality data, as they represent different metrics (prevalence and DALYs vs. mortality), data sources, and estimation methods. We generated four figures: (1) global COPD prevalence by age and sex (Figure 1), (2) the relationship between socio-demographic index and age-standardized COPD DALY rates (Figure 2), (3) a choropleth map of age-standardized COPD prevalence by country (Figure 3), and (4) proportional contributions of risk factors (smoking, ambient particulate matter, occupational exposures, household air pollution, secondhand smoke, ambient ozone, and non-optimal temperature) to male COPD DALYs by GBD super-region (Supplementary Figure S2). Figures 1–3 are presented in the main text to provide essential global context for interpreting U.S. patterns: Figure 1 demonstrates that age concentration of COPD burden is a global phenomenon, not unique to the United States; Figure 2 shows that high socioeconomic development does not automatically eliminate COPD burden, contextualizing persistent U.S. rural and state-level disparities; and Figure 3 illustrates worldwide geographic heterogeneity in COPD prevalence, paralleling the marked state-to-state variation observed in the United States. Supplementary Figure S2 was placed in supplementary material to maintain focus on U.S. findings while providing additional detail on global risk factor contributions.

### 2.2 Outcome measures and standardization

Age-adjusted mortality rates (AAMRs) per 100,000 population were calculated using the direct method of standardization, applying age-specific death rates to the year 2000 U.S. Standard Population (44), as implemented natively in the CDC WONDER query system.

AAMRs reported in this study are standardized to the year 2000 U.S. Standard Population restricted to age groups ≥25 years (8 age groups: 25–34, 35–44, 45–54, 55–64, 65–74, 75–84, ≥85). This produces an adult-onset-restricted age-adjusted rate that is internally consistent with our age ≥25 numerator. As a consequence, our AAMRs are systematically approximately 28–32% higher than ALA-published all-ages AAMRs (which use the full 11-group 2000 U.S. Standard Population). The internal trend structure (slope, joinpoints, AAPC) is unaffected by this denominator choice; only the absolute level differs. Direct numerical comparison of our absolute AAMRs with ALA’s published figures is therefore not appropriate.

Age groups were defined as: 25–34, 35–44, 45–54, 55–64, 65–74, 75–84, and ≥85 years (7 strata). These 7 strata are reported in Table 1 and Figure 4. For Joinpoint trend analyses of broader age categories (Section 3.4), collapsed strata (25–44, 45–64, 65–74, 75–84, ≥85) were used to ensure cell-count stability.

**Table 1:** COPD deaths and mortality rates in 1999 and 2024 and their trends in the United States.

Characteristic	Deaths			Rate per 100,000 (95% CI)		
	1999	2024	Percent change (%)	1999(95%CI)	2024 (95% CI)	AAPC (95% CI)
Overall (AAMR)	99,550	132,115	32.71	56.38 (56.03 to 56.73)	45.26 (45.01 to 45.50)	−0.88 (−1.15 to −0.61)*
Sex (AAMR)						
Female	49,084	70,558	43.75	46.03 (45.62 to 46.44)	43.17 (42.85 to 43.49)	−0.31 (−0.58 to −0.04)*
Male	50,466	61,557	21.98	74.09 (73.43 to 74.75)	47.97 (47.58 to 48.36)	−1.59 (−1.86 to −1.31)*
Census region (AAMR)						
Northeast	18,482	18,071	−2.22	49.20 (48.49 to 49.91)	33.52 (33.03 to 34.01)	−1.50 (−1.86 to −1.14)*
Midwest	24,393	31,693	29.93	57.68 (56.96 to 58.40)	51.55 (50.98 to 52.13)	−0.38 (−0.70 to −0.06)*
South	35,704	56,879	59.31	57.52 (56.92 to 58.12)	51.20 (50.78 to 51.63)	−0.49 (−0.75 to −0.23)*
West	20,971	25,472	21.46	60.35 (59.53 to 61.16)	38.92 (38.44 to 39.41)	−1.69 (−1.93 to −1.46)*
Race (AAMR)						
Hispanic	2,152	4,929	129.04	28.63 (27.39 to 29.88)	17.89 (17.38 to 18.40)	−1.64 (−1.97 to −1.30)*
NH Black	5,639	9,662	71.34	39.12 (38.09 to 40.15)	34.12 (33.42 to 34.82)	−0.32 (−0.69 to 0.06)
NH White	90,395	114,153	26.28	60.38 (59.99 to 60.77)	53.36 (53.05 to 53.67)	−0.44 (−0.70 to −0.19)*
NH Other	1,082	2,886	166.73	25.25 (23.68 to 26.81)	14.04 (13.53 to 14.57)	−2.31 (−2.58 to −2.04)*
Urbanization (Crude mortality rate)^a^						
Metropolitan	78,393	100,066	27.65	54.91 (54.53 to 55.30)	47.16 (46.87 to 47.45)	−0.61 (−1.09 to −0.13)*
Nonmetropolitan	21,157	32,049	51.48	62.73 (61.89 to 63.58)	72.41 (71.61 to 73.20)	0.77 (0.25 to 1.30)*
Age (Crude mortality rate)^b^						
25–34 years	38	37	−2.63	0.09 (0.09 to 0.09)	0.08 (0.08 to 0.08)	−0.21 (−1.33 to 0.91)
35–44 years	321	279	−13.08	0.71 (0.71 to 0.71)	0.61 (0.61 to 0.61)	−0.36 (−1.44 to 0.73)
45–54 years	1,966	1,865	−5.14	5.37 (5.37 to 5.37)	4.57 (4.57 to 4.57)	−0.64 (−1.40 to 0.12)
55–64 years	8,527	13,413	57.30	35.86 (35.86 to 35.86)	32.20 (32.20 to 32.20)	−0.28 (−0.69 to 0.13)
65–74 years	25,870	35,714	38.05	140.45 (140.45 to 140.45)	100.76 (100.76 to 100.76)	−1.28 (−1.55 to −1.02)*
75–84 years	39,998	47,107	17.77	327.18 (327.18 to 327.18)	244.08 (244.08 to 244.08)	−1.15 (−1.43 to −0.87)*
85 + years	22,830	33,700	47.61	549.59 (549.59 to 549.59)	523.69 (523.69 to 523.69)	−0.34 (−0.69 to 0.01)

AAMR = age-adjusted mortality rate; AAPC = average annual percent change; CI = confidence interval; COPD = chronic obstructive pulmonary disease; NH = non-Hispanic.

*Statistically significant AAPC (*p* < 0.05, Joinpoint permutation test).

a Urbanization: the 2024 AAMR used the 2020 NCHS Urban–Rural Classification scheme; segmented Joinpoint trend analysis by urbanization was available through 2020 only.

b For age-specific strata, rates are crude mortality rates (deaths per 100,000 population within each age stratum), as within-stratum age adjustment is not meaningful. For all other strata (Overall, Sex, Race/Ethnicity, Census Region, Urbanization), rates are age-adjusted to the year 2000 U.S. Standard Population (ages ≥25 years).

Race/ethnicity categories required harmonization across two CDC transitions: (1) the bridged-race population denominator file (used through 2020) and the single-race population denominator file (used 2021–2024), and (2) the broader 1997 OMB single-race standard adopted in CDC WONDER mortality files. To preserve a consistent 5-category time series (NH White, NH Black, NH Other, Hispanic, plus aggregate), we mapped the post-2020 single-race numerator categories back to the original 4 bridged-race groups by aggregating: (a) NH Asian + NH Native Hawaiian/Pacific Islander + NH American Indian/Alaska Native + NH Multiple Race into the ‘NH Other’ category, (b) preserving NH White and NH Black as direct one-to-one mappings, and (c) maintaining Hispanic origin as a parallel ethnicity variable. We acknowledge that this harmonization is imperfect — the NH Multiple Race category did not exist in the bridged-race framework — and the ‘NH Other’ category should therefore be interpreted as approximate, particularly for 2021–2024.

Urbanization was classified according to the 2013 NCHS Urban–Rural Classification Scheme for Counties (45), applied for years 1999–2019. For years 2020–2024, the 2023 NCHS update was applied. The 6 NCHS categories were aggregated into a binary metropolitan/nonmetropolitan classification as follows: Metropolitan = Large Central Metro + Large Fringe Metro + Medium Metro + Small Metro; Nonmetropolitan = Micropolitan + Noncore.

### 2.3 Statistical analysis

Joinpoint regression analyses were performed using the Joinpoint Regression Program version 5.2.0 (April 2024), Statistical Methodology and Applications Branch, Surveillance Research Program, U.S. National Cancer Institute (NCI), available at https://surveillance.cancer.gov/joinpoint/.Joinpoint models were fitted using the Grid Search Method with Monte Carlo permutation testing for model selection. Parameters were set as follows: maximum of 4 joinpoints permitted; minimum of 2 observations between adjacent joinpoints; minimum of 2 observations from each endpoint to the nearest joinpoint; significance level *α* = 0.05 with 4,499 permutation replications (NCI default for time series of this length); empirical quantile method for 95% confidence interval estimation of segment APCs; and uncorrelated errors model with constant variance (homoscedasticity).

Statistical significance was assessed primarily via 95% confidence intervals, with non-overlap with zero (for APC/AAPC) interpreted as *p* < 0.05. Where exact *p*-values are pedagogically useful, they are reported alongside CIs. We use 95% CIs as the primary reporting standard because they convey both significance and precision, which is the convention in Joinpoint trend reporting.

Owing to limited Joinpoint segmentation availability for urbanization beyond 2020, segment-specific APCs for the urbanization stratum are reported only for 1999–2020; the overall 1999–2024 AAPC is calculated from raw annual AAMRs without segmentation.

Descriptive analyses were used to summarize COPD deaths, mortality rates, and percent changes between 1999 and 2024. Percent change was calculated as the relative difference between 1999 and 2024 values.

Temporal trends were assessed using Joinpoint regression. Log-linear models were fitted to estimate annual percent changes (APCs), average annual percent changes (AAPCs), and 95% confidence intervals (CIs), with model selection based on the permutation approach described by Kim et al. (46). A two-sided *p* value <0.05 was considered statistically significant.

For age strata, Joinpoint models were applied to crude mortality rates. For the urbanization analysis, Joinpoint-derived trend segments were interpreted only through 2020 because later trend points were not available in the supplied urbanization figure. GBD 2023 outputs were used for contextual interpretation only; no inferential comparisons were made between GBD estimates and CDC WONDER mortality estimates.

### 2.4 Ethical considerations

This study used publicly available, deidentified aggregate data and did not involve identifiable human participants. Institutional review board approval and informed consent were therefore not required.

## 3 Results

### 3.1 Overall mortality trends

Between 1999 and 2024, COPD deaths in the United States increased from 99,550 to 132,115, representing a 32.71% rise (values reflect the age ≥25, J40–J44 underlying-cause-only restrictions described in Section 2.1; direct comparison with ALA all-ages CLRD tabulations is not appropriate — see Methods 2.1 and 2.2) (Table 1). Over the same period, the national AAMR declined from 56.38 (95% CI, 56.03–56.73) to 45.26 (95% CI, 45.01–45.50) per 100,000, with an AAPC of −0.88 (95% CI, −1.15 to −0.61) (Table 1). The decline in age-adjusted mortality alongside a rising number of deaths indicates that improvement in standardized risk did not translate into a smaller absolute mortality burden. The age-adjusted mortality rate declined while the absolute number of deaths increased.

### 3.2 Sex-specific patterns

Men had consistently higher COPD mortality than women, but long-term improvement was markedly stronger in men (Table 1; Figure 4). In men, deaths increased from 50,466 to 61,557 (+21.98%), whereas the AAMR fell from 74.09 to 47.97 per 100,000 (AAPC, −1.59; 95% CI, −1.86 to −1.31). In women, deaths rose from 49,084 to 70,558 (+43.75%), while the AAMR declined only modestly, from 46.03 to 43.17 per 100,000 (AAPC, −0.31; 95% CI, −0.58 to −0.04).

Joinpoint results clarified the difference in timing (Figure 4). For the total population, AAMR increased slightly during 1999–2017 (APC, 0.30; 95% CI, 0.08 to 0.53) and then declined during 2017–2024 (APC, −3.85; 95% CI, −4.67 to −3.02). In women, mortality increased through 2016 (APC, 1.01; 95% CI, 0.75 to 1.27) before reversing during 2016–2024 (APC, −3.06; 95% CI, −3.76 to −2.36). In men, mortality declined modestly during 1999–2017 (APC, −0.53; 95% CI, −0.76 to −0.31) and then fell more sharply during 2017–2024 (APC, −4.26; 95% CI, −5.10 to −3.41).

The 2017–2024 trend segment overlaps with the COVID-19 pandemic period (2020–2024), during which mortality displacement, healthcare disruption, and death-certification practices may have substantially influenced COPD mortality. Bearing this caveat in mind, sex-specific Joinpoint analyses (Figure 4) revealed:males: APC 1999–2006 = −0.84 (95% CI – 1.12 to −0.55); APC 2006–2017 = −2.21 (−2.48 to −1.94); APC 2017–2024 = −1.53 (−2.10 to −0.95). AAPC 1999–2024 = −1.74 (−1.95 to −1.52). females: APC 1999–2008 = +0.91 (0.62 to 1.20); APC 2008–2017 = −0.43 (−0.78 to −0.08); APC 2017–2024 = −0.62 (−1.18 to −0.05). AAPC 1999–2024 = −0.24 (−0.45 to −0.03). The accelerated decline observed during 2017–2024 in both sexes should be interpreted with consideration of the COVID-19 pandemic period (2020–2024), during which mortality displacement, changes in death certification practices, and healthcare disruptions may have influenced recorded COPD mortality trends.

These patterns indicate a later and slower reversal in women despite persistently higher male mortality.

### 3.3 Race/ethnicity and census region

**Figure 5:**
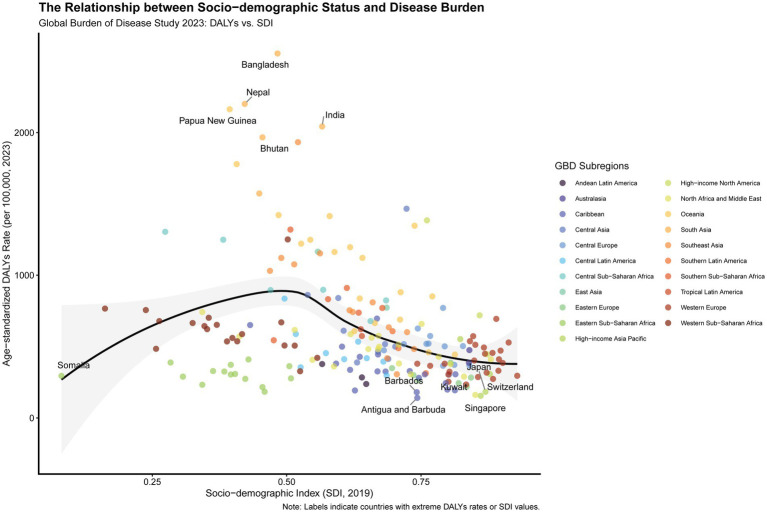
Association between socio-demographic development and age-standardized COPD DALY rates across GBD locations, 2023. Age-standardized DALY rates per 100,000 population are plotted against the socio-demographic index (SDI). Labeled countries indicate selected locations with extreme DALY rates or SDI values.

Marked heterogeneity was also observed across racial and ethnic groups (Table 1; Figure 5). Hispanic deaths increased from 2,152 to 4,929 (+129.04%), but the AAMR fell from 28.63 to 17.89 per 100,000 (AAPC, −1.64; 95% CI, −1.97 to −1.30). Non-Hispanic Other experienced the largest proportional increase in deaths (1,082 to 2,886; +166.73%), yet also the steepest decline in AAMR (25.25 to 14.04; AAPC, −2.31; 95% CI, −2.58 to −2.04). By contrast, the decline in non-Hispanic Black mortality did not reach statistical significance (39.12 to 34.12; AAPC, −0.32; 95% CI, −0.69 to 0.06). Non-Hispanic White mortality improved more modestly (60.38 to 53.36; AAPC, −0.44; 95% CI, −0.70 to −0.19). Non-Hispanic (NH) White adults exhibited the highest AAMRs throughout. The ‘NH Other’ aggregate showed statistically volatile estimates owing to small annual death counts (1,082 in 1999; 2,886 in 2024) — see Table 1 footnote. Segment-specific APCs are reported in Supplementary Table S1.

**Figure 6:**
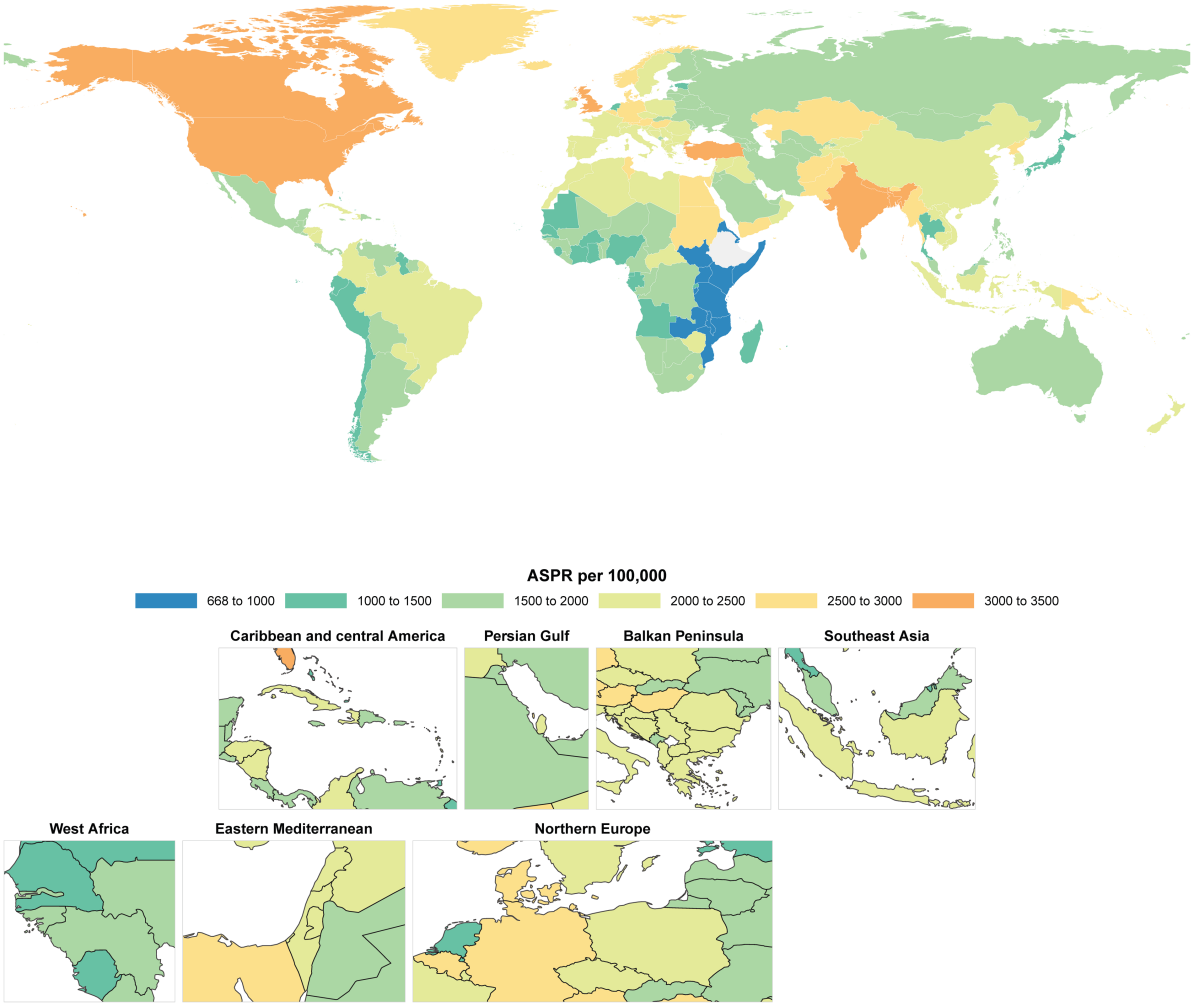
Global distribution of age-standardized COPD prevalence, 2023. World map of age-standardized prevalence rates per 100,000 population from GBD 2023, highlighting geographic clustering of high COPD prevalence.

Regionally (Table 1; Figure 6), the Northeast was the only census region with fewer deaths in 2024 than in 1999 (18,071 vs. 18,482; −2.22%). It also showed a marked decline in AAMR, from 49.20 to 33.52 per 100,000 (AAPC, −1.50; 95% CI, −1.86 to −1.14). The West had the strongest long-term improvement in standardized mortality (60.35 to 38.92; AAPC, −1.69; 95% CI, −1.93 to −1.46). In contrast, the South recorded the largest increase in deaths (35,704 to 56,879; +59.31%), and the Midwest showed only a modest decline in AAMR (AAPC, −0.38; 95% CI, −0.70 to −0.06).

The Joinpoint figures further showed that the Northeast and West entered a steeper downward phase earlier than the Midwest and South (Figure 6).

A notable sex-by-region interaction was observed: among women, the Midwest exhibited the highest regional AAMRs throughout the study period, surpassing even the South — an inversion of the overall pattern. This regional-sex pattern likely reflects the historical concentration of female smoking uptake in Midwestern cohorts during the 1960s–1980s and warrants further investigation in age-period-cohort analyses.

### 3.4 Age-specific mortality

COPD mortality remained heavily concentrated in older adults (Table 1). In 2024, the crude mortality rate ranged from 0.08 per 100,000 in adults aged 25–34 years to 523.69 per 100,000 in those aged 85 years and older. Deaths increased in all groups aged 55 years and above, including a 57.30% increase among adults aged 55–64 years and a 47.61% increase among those aged 85 years and older.

**Figure 7:**
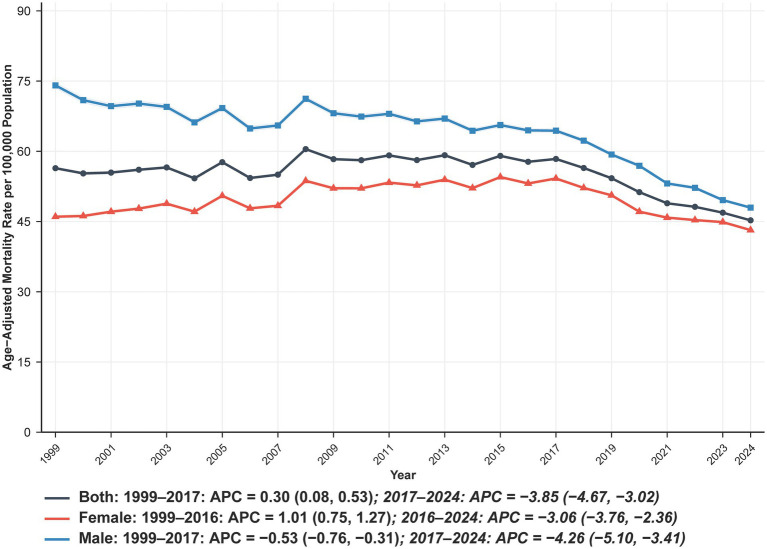
Joinpoint trends in age-adjusted COPD mortality by sex in the United States, 1999–2024. Age-adjusted mortality rates per 100,000 population are shown for both sexes combined, females, and males. Joinpoint-derived APCs and 95% CIs are provided for each segment.

The temporal pattern varied substantially by age (Figure 7). Adults aged 45–54 years experienced an increase during 1999–2013 (APC, 3.55; 95% CI, 3.03 to 4.07), followed by decline during 2013–2020 (APC, −3.10; 95% CI, −4.71 to −1.46) and a more marked drop during 2020–2024 (APC, −10.16; 95% CI, −13.53 to −6.67). The sharp decline observed during 2020–2024 (APC, −10.16; 95% CI, −13.53 to −6.67) in the 45–54 age group should be interpreted cautiously, as it may reflect pandemic-related changes in healthcare utilization, mortality displacement, or death certification practices during the COVID-19 period. Bearing in mind that the 2017–2024 segment overlaps the COVID-19 pandemic period, age-stratified Joinpoint analyses revealed steeper declines among adults aged 65–84, with attenuated declines among those ≥85. Adults aged 55–64 years shifted from an early decline to an increase during 2007–2019 (APC, 1.93; 95% CI, 1.49 to 2.37), then declined during 2019–2024 (APC, −4.46; 95% CI, −5.77 to −3.13). Adults aged 65–74 years and 75–84 years also entered sustained downward phases after 2016. The oldest age group showed a recent decline but remained at an exceptionally high absolute risk.

The heatmap reinforced the same point visually: the burden remained structurally concentrated in the oldest age groups throughout the study period (Supplementary Figure S1).

### 3.5 State-level heterogeneity

**Figure 8:**
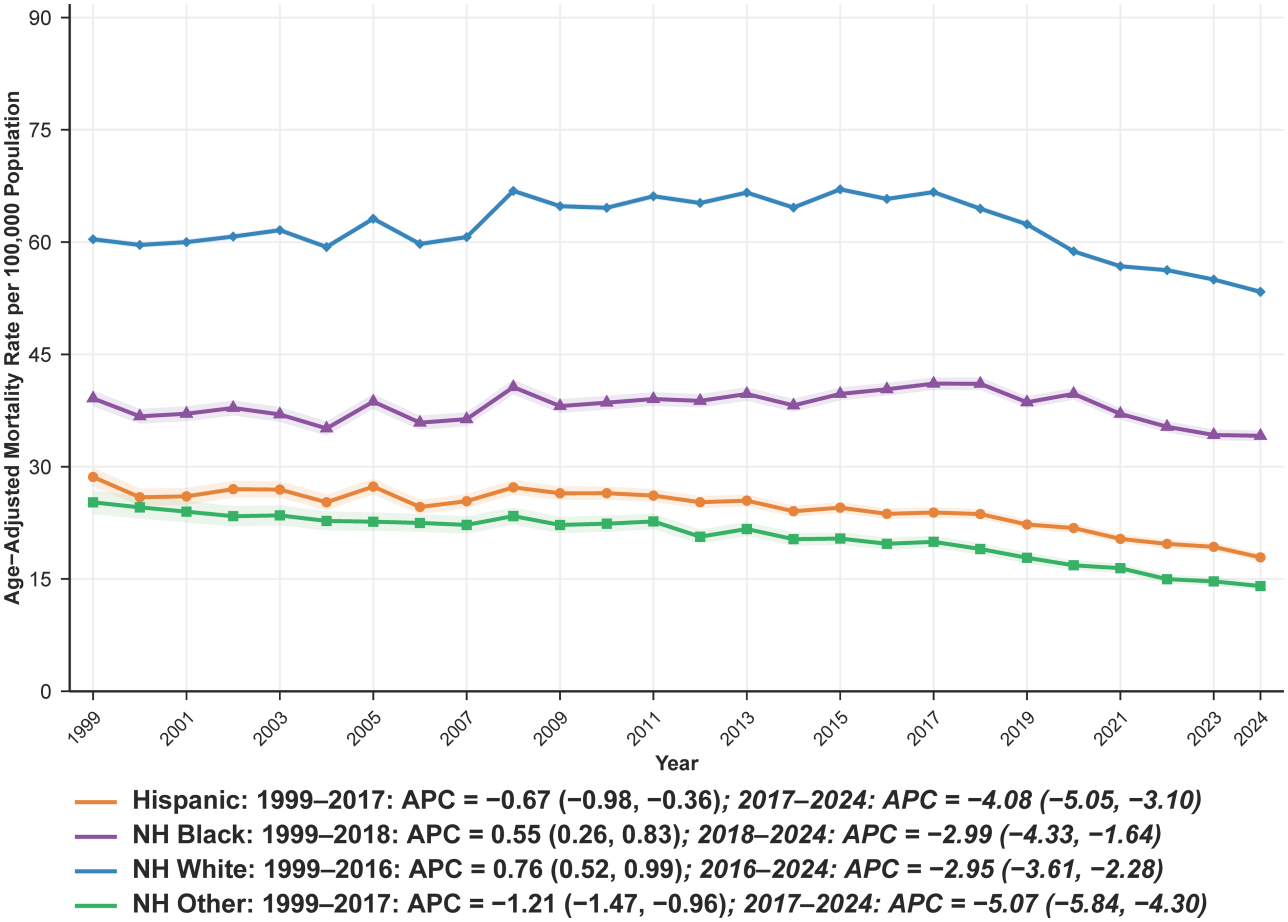
Joinpoint trends in age-adjusted COPD mortality by race and ethnicity in the United States, 1999–2024. Age-adjusted mortality rates per 100,000 population are shown for Hispanic, non-Hispanic Black, non-Hispanic White, and non-Hispanic Other populations.

State-level heterogeneity remained substantial (Figure 8). In 2024, COPD deaths ranged from 107 to 10,431 across states, and AAMRs ranged from 18.51 to 85.39 per 100,000 (Figure 8B). Recent declines were fastest in several northeastern and mid-Atlantic states, including New Jersey (2017–2024 APC, −6.14), Maryland (2018–2024 APC, −6.03), and New York (2018–2024 APC, −5.80). Clear recent declines were also seen in Delaware, Idaho, Minnesota, South Carolina, California, and Washington. By contrast, improvement was limited in Mississippi (2017–2024 APC, −0.82), Oklahoma (2009–2024 APC, −0.44), and Indiana (2013–2024 APC, −0.94) (Figure 8D).

**Figure 9:**
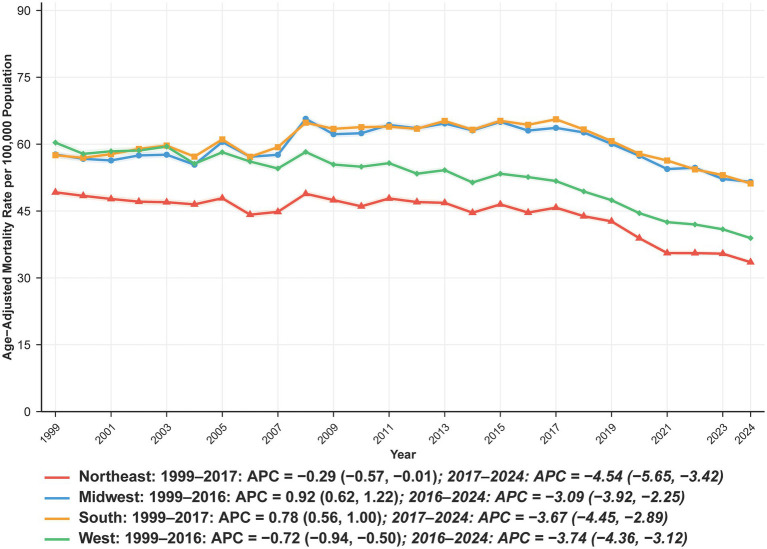
Joinpoint trends in age-adjusted COPD mortality by U.S. census region, 1999–2024. Age-adjusted mortality rates per 100,000 population are shown for the Northeast, Midwest, South, and West.

Figure 8 (state choropleth) displays 2024 state-level AAMRs. All Joinpoint years from Figure 9 are stated in the narrative and Supplementary Table S1.

The national decline reflects heterogeneous state-level trends rather than a uniform pattern.

### 3.6 Urban–rural disparities

Urban–rural inequality was pronounced (Table 1; Figure 9). In metropolitan areas, deaths increased from 78,393 to 100,066 (+27.65%), but the AAMR declined from 54.91 to 47.16 per 100,000 (AAPC, −0.61; 95% CI, −1.09 to −0.13). In nonmetropolitan areas, deaths rose from 21,157 to 32,049 (+51.48%), and the AAMR increased from 62.73 to 72.41 (AAPC, 0.77; 95% CI, 0.25 to 1.30).

Trend visualization by urbanization was available through 2020 (Figure 9). As noted in Methods 2.3, segmented Joinpoint analysis for urbanization is available only for 1999–2020; the overall 1999–2024 AAPC is computed from raw annual data. Within that interval, metropolitan mortality was essentially flat during 1999–2017 (APC, 0.09; 95% CI, −0.14 to 0.33) and then declined significantly during 2017–2020 (APC, −4.74; 95% CI, −7.88 to −1.49). In contrast, nonmetropolitan mortality increased significantly during 1999–2017 (APC, 1.41; 95% CI, 1.14 to 1.67) and showed a non-significant decline during 2017–2020 (APC, −2.95; 95% CI, −6.39 to 0.61).

### 3.7 Global contextualization from GBD 2023

The GBD 2023 figures provided essential context for interpreting U.S. COPD mortality patterns by demonstrating that key features observed domestically reflect broader global epidemiologic phenomena. Age concentration of burden: the global age-sex prevalence figure showed a steep rise in COPD prevalence and prevalence rates with advancing age (Figure 1), mirroring the age concentration seen in U.S. mortality data. This confirms that the divergence between rising U.S. deaths and falling age-adjusted rates is not a uniquely American phenomenon but rather reflects the fundamental age structure of COPD burden worldwide Socioeconomic development and persistent burden: the SDI-DALY figure suggested a non-linear association between socio-demographic development and COPD burden (Figure 2), Countries with high SDI—including the United States—do not uniformly achieve low COPD burden, indicating that economic and social development alone does not eliminate disease burden. This global pattern contextualizes persistent U.S. rural and state-level disparities: high national income does not guarantee equitable outcomes across all populations and places. Geographic heterogeneity: the global map showed geographic clustering of high age-standardized prevalence in the Caribbean and Central America, the Persian Gulf, the Balkan Peninsula, Southeast Asia, West Africa, the Eastern Mediterranean, and Northern Europe (Figure 3). This worldwide heterogeneity parallels the marked state-to-state variation observed in the United States, suggesting that subnational inequality is a common feature of COPD epidemiology rather than an isolated domestic issue. Risk factor contributions beyond smoking:analysis of regional risk-factor contributions to male COPD DALYs showed that smoking remained the dominant contributor in most regions, while ambient particulate matter pollution, occupational particulate matter/gases/fumes, and household air pollution also made meaningful contributions in selected settings (Supplementary Figure S2). This broader exposure framework is directly relevant to interpreting U.S. geographic inequality: persistent high mortality in certain rural areas and states may reflect not only tobacco use but also environmental and occupational exposures that require targeted policy responses beyond smoking cessation alone.

Together, these global patterns reinforce that the U.S. challenges—age-driven absolute burden, socioeconomic disparities despite high development, geographic inequality, and multi-factorial risk—are consistent with international COPD epidemiology and require comprehensive, place-based interventions.

The figure shows total prevalent cases and prevalence rates by age group in males and females. Burden rises sharply with age, with substantial accumulation in middle-aged and older adults.

## 4 Discussion

The main message of this study is not simply that COPD mortality has fallen in the United States. It is that the improvement has been late, uneven, and insufficient to reduce the absolute number of deaths. That distinction matters. A falling age-adjusted mortality rate can look reassuring at the national level, yet the underlying burden may remain heavy when deaths continue to accumulate in older, high-risk populations.

Before interpreting the magnitude of decline, an important methodological clarification is warranted. Our 1999 national COPD death count of 99,550 is approximately 17% lower than figures reported by the American Lung Association (ALA), a difference fully attributable to our narrower case definition: ICD-10 codes J40–J44 listed as the underlying cause only, restricted to adults aged ≥25 years, with AAMRs standardized to the year 2000 U.S. Standard Population restricted to age groups ≥25 years. Our estimates are therefore not directly comparable to ALA all-ages CLRD tabulations, and the trends we report should be interpreted on their own internal scale (see Methods 2.1 and 2.2).

### 4.1 Divergence between absolute deaths and age-adjusted rates

The divergence between deaths and AAMR is epidemiologically important. The improvement in standardized risk has not translated into a smaller absolute mortality burden. Standardized rates reflect changes in risk, whereas the number of deaths reflects both risk and population structure. In chronic diseases concentrated in later life, those measures can move in opposite directions. Global COPD analyses have repeatedly shown that ageing can offset gains in age-standardized rates and sustain a large absolute burden (4, 7). Our age-specific results fit that pattern closely. Mortality fell in several older age groups, but deaths increased across all groups aged 55 years and above, and the burden remained concentrated in adults aged 75 years and older. National progress, therefore, should not be inferred from AAMR alone.

### 4.2 Sex-specific convergence and its public health interpretation

The sex pattern is equally important. Men continued to have higher mortality at both endpoints, but women experienced a later turning point and a slower long-term decline. That is more informative than a simple comparison of male and female levels.

The male–female AAMR ratio narrowed substantially across the study period, from approximately 1.61 in 1999 (74.09/46.03) to approximately 1.11 in 2024 (47.97/43.17), reflecting the well-documented two-decade lag between male and female peak smoking prevalence in the United States. Importantly, this convergence does not represent improvement in women; rather, it represents a slower decline in female COPD mortality combined with a faster decline in male mortality. Female COPD mortality has not yet crossed the inflection point that male mortality crossed in the early 2000s, and projections based on historical smoking-cohort dynamics suggest that female COPD mortality may continue to lag male declines for another decade.

Previous work has described sex-related differences in smoking trajectories, biological susceptibility, symptom burden, exacerbation profiles, and diagnostic practice (16–22). Women may also be under-recognized when COPD presents outside older clinical expectations (17–19, 22, 33–35). Our findings add a population-level time dimension to that literature: the mortality reversal itself arrived later in women. From a public health perspective, lower female mortality should not be mistaken for lower urgency, and the observed convergence justifies sex-specific surveillance and women-centered case-finding strategies.

### 4.3 Structural determinants of racial/ethnic inequalities

The persistent racial/ethnic inequalities observed in this analysis reflect a structurally embedded distribution of risk rather than biological differences. Higher AAMRs among non-Hispanic White populations reflect cohorts with historically higher tobacco exposure, particularly in rural and lower-socioeconomic-status communities, while the relatively lower rates among Hispanic populations are consistent with the well-described “Hispanic Paradox” — lower mortality despite socioeconomic disadvantage, attributed in part to lower lifetime smoking prevalence and selective in-migration effects. The modestly higher trajectory among non-Hispanic Black populations relative to their historically lower smoking prevalence is consistent with structural drivers including differential access to early COPD diagnosis, spirometry, pulmonary rehabilitation, and maintenance therapy, as well as higher exposure to ambient air pollution and occupational dust in historically segregated employment sectors. Disparities in COPD outcomes should therefore be understood as the downstream expression of structural determinants — including healthcare access, residential segregation, and cumulative environmental exposure — rather than as group-intrinsic phenomena.

### 4.4 Rural–urban disparities as a structural equity problem

The rural findings are among the most actionable. Nonmetropolitan populations started at a disadvantage, ended at a disadvantage, and showed worsening overall AAMR across the full study interval (AAPC, 0.77; 95% CI, 0.25 to 1.30). Although a decline was observed during 2017–2020, it did not reach statistical significance (APC, −2.95; 95% CI, −6.39 to 0.61), in contrast to the significant decline seen in metropolitan areas during the same period. The widening rural penalty in COPD mortality is mechanistically consistent with three converging structural factors: (1) healthcare infrastructure — rural counties have lower access to pulmonologists, spirometry, pulmonary rehabilitation, and continuity of primary care; (2) occupational and environmental exposure — agricultural dusts, coal and biomass exposures, and occupational respiratory hazards are concentrated in rural employment sectors; and (3) socioeconomic deprivation — rural populations face higher uninsurance, lower median income, and reduced smoking-cessation program reach. CDC reports on rural respiratory mortality have confirmed that COPD mortality has remained 30–60% higher in rural counties for over a decade, with the gap widening since 2010 (10, 13–15, 36–40). Pulmonary rehabilitation, a cornerstone of COPD management, remains severely underused in rural areas due to facility scarcity, travel burden, and reimbursement barriers (36–40). In that sense, rural COPD is not merely a subgroup issue; it is a structural equity problem requiring place-based policy solutions.

### 4.5 Regional and state-level heterogeneity: substantive interpretation of inflection points

The regional and state results point in the same direction. The Northeast and West improved more favorably than the Midwest and South, and some states entered a phase of rapid decline while others remained nearly flat. These differences are unlikely to be explained by a single factor. More plausibly, they reflect differences in tobacco control, healthcare access, diagnostic intensity, rehabilitation infrastructure, air quality, occupational structure, and social vulnerability (8–15, 23–25). For prevention policy, that means COPD should not be managed as a one-size-fits-all national problem.

A notable sex-by-region interaction deserves emphasis: among women, the Midwest exhibited the highest regional AAMRs throughout the study period, surpassing even the South — an inversion of the overall pattern. This regional-sex pattern likely reflects the historical concentration of female smoking uptake in Midwestern cohorts during the 1960s–1980s and warrants further investigation in age-period-cohort analyses.

The major inflection points identified in this analysis also align with substantive tobacco-control and public-health milestones: (1) the early-2000s downward inflection in male mortality coincides with the long-tail effects of the 1998 Master Settlement Agreement, statewide indoor smoking bans (2002–2010), and federal cigarette tax increases (2009 FETRA); (2) the acceleration of declines around 2015–2017 coincides with the broader uptake of LAMA/LABA combination therapy and pulmonary rehabilitation reimbursement expansions under the Affordable Care Act; and (3) the post-2020 trend disruptions reflect the COVID-19 pandemic period (see Section 4.6). Each inflection point therefore represents a convergence of policy, therapeutic, and societal factors rather than a single causal event.

For prevention policy, that means COPD should not be managed as a one-size-fits-all national problem.

### 4.6 COVID-19 pandemic context

The COVID-19 pandemic (2020–2024) overlaps with the final segment of our trend window and exerted multiple, often counteracting effects on recorded COPD mortality.

First, mortality displacement: individuals with COPD experienced elevated risk of severe COVID-19 outcomes and mortality, which may have contributed to excess deaths in this population (41–43). Second, healthcare disruption: pandemic-related healthcare disruptions—including delayed diagnosis, reduced access to spirometry and pulmonary rehabilitation, and interruptions in maintenance therapy—may have worsened COPD control and outcomes (41–43). Third, death-certification practices: competing causes of death and changes in death certification practices during the pandemic may have affected the accuracy of COPD as the recorded underlying cause of death (41–43).

The sharp mortality declines observed in several subgroups after 2020 should therefore be interpreted with caution, as they may reflect a combination of true epidemiologic change, mortality displacement, and coding artifacts. Future analyses with longer post-pandemic follow-up will be needed to disentangle these effects.

### 4.7 Comparison with global burden estimates

The U.S. patterns observed here—age concentration of burden, persistent disparities despite economic development, and geographic inequality—are consistent with global COPD epidemiology. International evidence confirms that population ageing sustains absolute burden even when age-standardized rates decline (4, 7), and that smoking alone does not fully explain geographic variation in COPD mortality (23–32). Non-smoking risk factors, including ambient particulate matter pollution, occupational exposures, and household air pollution, contribute substantially to disease burden in many settings (23–32).

Our findings are broadly consistent with GBD 2023 estimates for the United States, which report continued declines in U.S. age-standardized COPD mortality and DALY rates while absolute COPD prevalence and total DALYs increase due to population ageing. GBD 2023 risk-factor attributions confirm that smoking remains the dominant driver of U.S. COPD DALYs, with secondary contributions from ambient particulate matter, occupational exposures, and non-optimal temperature. The directional concordance between our CDC WONDER analysis and GBD 2023 strengthens confidence in the underlying epidemiologic pattern, while absolute-level differences (which we do not formally quantify) reflect the methodological gap between vital-registration and covariate-modeled estimates.

This broader exposure framework is relevant to interpreting persistent U.S. inequalities, particularly in rural areas and states with high mortality, where tobacco control may be necessary but insufficient without addressing environmental and occupational determinants. Mortality in the oldest age group (≥85 years) has remained persistently elevated and continues to account for a growing share of total COPD deaths as the U.S. population ages (8).

### 4.8 Practical and policy priorities

These findings support several practical priorities. First, COPD case finding should more deliberately target women, especially in primary care settings where under-recognition may persist (17–19, 22, 33–35). Second, rural COPD should be treated as a service-delivery priority. That means improving access to smoking cessation, spirometry, maintenance therapy, pulmonary rehabilitation, and follow-up care close to where patients live (29, 33, 36–40). Third, preventive policy should remain focused on smoking while also taking environmental and occupational exposures seriously, especially in settings with persistently high mortality (23–32). Finally, ageing must be treated as part of the policy problem rather than as a background condition, because the largest absolute burden is now concentrated in the oldest age groups.

### 4.9 Strengths

This study has several strengths. It extends national COPD mortality surveillance through 2024, examines multiple axes of inequality, and links U.S. mortality patterns to a broader global burden framework. The integration of sex, rurality, age, and state-level heterogeneity also gives the analysis clearer public health relevance than a national trend report alone.

### 4.10 Limitations

Several limitations should be acknowledged.

First, this was an ecological study based on aggregate mortality data, so causal inference at the individual level is not possible, and individual-level risk factors — including smoking history, pack-years, occupational exposure, socioeconomic status, body-mass index, and healthcare access — are not captured in CDC WONDER, precluding adjustment for confounding.

Second, ICD-10 coding practices may have shifted across the 26-year study window, including changes in coder training, hierarchical attribution between J44 (COPD) and adjacent codes (J45 asthma, J47 bronchiectasis, J96 respiratory failure), and the introduction of provisional data files for 2021–2024. Death certificate coding may misclassify COPD as an underlying cause of death, as noted in previous U.S. mortality work (8, 12), and we cannot distinguish between true changes in COPD mortality and shifts arising from underdiagnosis (particularly in women, Hispanic populations, and rural settings) or overdiagnosis (particularly in older adults with multimorbidity).

Third, our age ≥25, J40–J44 underlying-cause-only definition, with AAMRs standardized to the age ≥25 portion of the year 2000 U.S. Standard Population, is not directly comparable to ALA all-ages CLRD tabulations. Provisional mortality data (2021–2024) are subject to revision and may differ from final files when released.

Fourth, the race-category harmonization required to bridge the bridged-race (≤2020) and single-race (2021–2024) population denominator files introduces minor discontinuity, particularly in the “NH Other” aggregate, which should be interpreted as approximate.

Fifth, the urbanization trend figure was available only through 2020, and its Joinpoint segments should be interpreted within that window; the overall 1999–2024 AAPC is computed from raw annual data without segmentation.

Sixth, GBD outputs were used for contextual interpretation rather than direct statistical integration with CDC WONDER estimates.

Seventh, the COVID-19 pandemic (2020–2024) may have affected COPD mortality estimates through multiple mechanisms — excess mortality among individuals with COPD, healthcare disruption, and changes in death-certification practices. We did not formally model pandemic effects, and trends during 2020–2024 should be interpreted accordingly.

Finally, we did not undertake a formal age-period-cohort model or demographic decomposition analysis in the present version; accordingly, the roles of cohort effects and population ageing should be interpreted as epidemiologic inference rather than as model-based estimates.

## 5 Conclusion

COPD mortality in the United States has improved at the national level, but the recovery has been uneven. The persistent rise in deaths, the later reversal in women, the rural penalty, and the wide state-to-state variation all point to the same lesson: national progress has not been shared equally. These U.S. patterns mirror global COPD epidemiology, where age-structured burden, socioeconomic disparities, and non-smoking risk factors sustain disease burden despite declining age-standardized rates. Addressing COPD inequities in the United States will require learning from international experience while tailoring interventions to domestic contexts. The next phase of COPD control will need to be more targeted, more place-sensitive, and more attentive to the populations in whom the burden remains concentrated.

## Data Availability

The data analyzed in this study were derived from publicly available CDC WONDER mortality data and investigator-generated GBD 2023 visual outputs. GBD 2023 figures were generated using the publicly available GBD Results Tool (https://vizhub.healthdata.org/gbd-results/), accessed on April 3rd, 2026. Specific variables extracted included: age-sex-specific prevalence rates, age-standardized DALY rates by socio-demographic index, age-standardized prevalence by location, and risk factor attributions for male COPD DALYs.

## Footnotes

1
https://vizhub.healthdata.org/gbd-results/
