# Supplementary material for: Declining age-adjusted mortality but persistent inequalities in chronic obstructive pulmonary disease in the United States, 1999–2024: a CDC WONDER analysis contextualized by GBD 2023
Source: Front Public Health. 2026 Jun 18;14:1852919. doi: 10.3389/fpubh.2026.1852919 (PMC13323240; doi:10.3389/fpubh.2026.1852919)
Supplement: Supplementary file 1 [file Data_Sheet_1.pdf]

**Supplementary Table S1: Complete Joinpoint segmentation, segment-specific APCs, and 95% CIs for all demographic strata, 1999–2024**

| Demographic Stratum | Subgroup | Segment/Overall | Years | APC / AAPC (%) | 95% CI (%) | P Value | Trend Direction |
|---|---|---|---|---|---|---|---|
| Sex | Both | Segment 1 | 1999–2017 | 0.30 | (0.08, 0.53) | 0.010 | Significant Increase |
| | Both | Segment 2 | 2017–2024 | -3.85 | (-4.67, -3.02) | <0.001 | Significant Decrease |
| | | AAPC | 1999–2024 | -0.88 | (-1.15, -0.61) | <0.001 | Significant Decrease |
| | Male | Segment 1 | 1999–2017 | -0.53 | (-0.76, -0.31) | <0.001 | Significant Decrease |
| | | Segment 2 | 2017–2024 | -4.26 | (-5.10, -3.41) | <0.001 | Significant Decrease |
| | | AAPC | 1999–2024 | -1.59 | (-1.86, -1.31) | <0.001 | Significant Decrease |
| | Female | Segment 1 | 1999–2016 | 1.01 | (0.75, 1.27) | <0.001 | Significant Increase |
| | | Segment 2 | 2016–2024 | -3.06 | (-3.76, -2.36) | <0.001 | Significant Decrease |
| | | AAPC | 1999–2024 | -0.31 | (-0.58, -0.04) | 0.025 | Significant Decrease |
| Age Group (Years) | 25-34 years | Segment 1 | 1999–2024 | -0.21 | (-1.33, 0.91) | 0.696 | Stable |
| | 25-34 years | AAPC | 1999–2024 | -0.21 | (-1.33, 0.91) | 0.696 | Stable |
| | 35-44 years | Segment 1 | 1999–2003 | 7.73 | (0.84, 15.09) | 0.029 | Significant Increase |
| | | Segment 2 | 2003–2024 | -1.83 | (-2.37, -1.29) | <0.001 | Significant Decrease |
| | | AAPC | 1999–2024 | -0.36 | (-1.44, 0.73) | 0.516 | Stable |
| | 45-54 years | Segment 1 | 1999–2013 | 3.55 | (3.03, 4.07) | <0.001 | Significant Increase |
| | | Segment 2 | 2013–2020 | -3.10 | (-4.71, -1.46) | <0.001 | Significant Decrease |
| | | Segment 3 | 2020–2024 | -10.16 | (-13.53, -6.67) | <0.001 | Significant Decrease |
| | | AAPC | 1999–2024 | -0.64 | (-1.40, 0.12) | 0.100 | Stable |
| | 55-64 years | Segment 1 | 1999–2007 | -0.90 | (-1.75, -0.05) | 0.040 | Significant Decrease |
| | | Segment 2 | 2007–2019 | 1.93 | (1.49, 2.37) | <0.001 | Significant Increase |
| | | Segment 3 | 2019–2024 | -4.46 | (-5.77, -3.13) | <0.001 | Significant Decrease |
| | | AAPC | 1999–2024 | -0.28 | (-0.69, 0.13) | 0.175 | Stable |
| | 65-74 years | Segment 1 | 1999–2016 | -0.47 | (-0.72, -0.22) | <0.001 | Significant Decrease |

| | | | | | | | |
|---|---|---|---|---|---|---|---|
| | | Segment 2 | 2016–2024 | -2.99 | (-3.66, -2.31) | <0.001 | Significant Decrease |
| | | AAPC | 1999–2024 | -1.28 | (-1.55, -1.02) | <0.001 | Significant Decrease |
| | 75-84 years | Segment 1 | 1999–2016 | 0.26 | (0.00, 0.52) | 0.047 | Significant Increase |
| | | Segment 2 | 2016–2024 | -4.09 | (-4.81, -3.36) | <0.001 | Significant Decrease |
| | | AAPC | 1999–2024 | -1.15 | (-1.43, -0.87) | <0.001 | Significant Decrease |
| | 85+ years | Segment 1 | 1999–2017 | 0.93 | (0.64, 1.22) | <0.001 | Significant Increase |
| | | Segment 2 | 2017–2024 | -3.54 | (-4.59, -2.48) | <0.001 | Significant Decrease |
| | | AAPC | 1999–2024 | -0.34 | (-0.69, 0.01) | 0.055 | Stable |
| Race/Ethnicity | Hispanic | Segment 1 | 1999–2017 | -0.67 | (-0.98, -0.36) | <0.001 | Significant Decrease |
| | Hispanic | Segment 2 | 2017–2024 | -4.08 | (-5.05, -3.10) | <0.001 | Significant Decrease |
| | | AAPC | 1999–2024 | -1.64 | (-1.97, -1.30) | <0.001 | Significant Decrease |
| | NH Black | Segment 1 | 1999–2018 | 0.55 | (0.26, 0.83) | <0.001 | Significant Increase |
| | | Segment 2 | 2018–2024 | -2.99 | (-4.33, -1.64) | <0.001 | Significant Decrease |
| | | AAPC | 1999–2024 | -0.32 | (-0.69, 0.06) | 0.098 | Stable |
| | NH White | Segment 1 | 1999–2016 | 0.76 | (0.52, 0.99) | <0.001 | Significant Increase |
| | | Segment 2 | 2016–2024 | -2.95 | (-3.61, -2.28) | <0.001 | Significant Decrease |
| | | AAPC | 1999–2024 | -0.44 | (-0.70, -0.19) | <0.001 | Significant Decrease |
| | NH Other | Segment 1 | 1999–2017 | -1.21 | (-1.47, -0.96) | <0.001 | Significant Decrease |
| | | Segment 2 | 2017–2024 | -5.07 | (-5.84, -4.30) | <0.001 | Significant Decrease |
| | | AAPC | 1999–2024 | -2.31 | (-2.58, -2.04) | <0.001 | Significant Decrease |
| Census Region | Northeast | Segment 1 | 1999–2017 | -0.29 | (-0.57, -0.01) | 0.041 | Significant Decrease |
| | Northeast | Segment 2 | 2017–2024 | -4.54 | (-5.65, -3.42) | <0.001 | Significant Decrease |
| | | AAPC | 1999–2024 | -1.50 | (-1.86, -1.14) | <0.001 | Significant Decrease |
| | Midwest | Segment 1 | 1999–2016 | 0.92 | (0.62, 1.22) | <0.001 | Significant Increase |
| | | Segment 2 | 2016–2024 | -3.09 | (-3.92, -2.25) | <0.001 | Significant Decrease |
| | | AAPC | 1999–2024 | -0.38 | (-0.70, -0.06) | 0.021 | Significant Decrease |

| | | | | | | | |
|---|---|---|---|---|---|---|---|
| | South | Segment 1 | 1999–2017 | 0.78 | (0.56, 1.00) | <0.001 | Significant Increase |
| | | Segment 2 | 2017–2024 | -3.67 | (-4.45, -2.89) | <0.001 | Significant Decrease |
| | | AAPC | 1999–2024 | -0.49 | (-0.75, -0.23) | <0.001 | Significant Decrease |
| | West | Segment 1 | 1999–2016 | -0.72 | (-0.94, -0.50) | <0.001 | Significant Decrease |
| | | Segment 2 | 2016–2024 | -3.74 | (-4.36, -3.12) | <0.001 | Significant Decrease |
| | | AAPC | 1999–2024 | -1.69 | (-1.93, -1.46) | <0.001 | Significant Decrease |
| Urbanization Status | Metropolitan | Segment 1 | 1999–2017 | 0.09 | (-0.14, 0.33) | 0.423 | Stable |
| | Metropolitan | Segment 2 | 2017–2020 | -4.74 | (-7.88, -1.49) | 0.007 | Significant Decrease |
| | | AAPC | 1999–2020 | -0.61 | (-1.09, -0.13) | 0.012 | Significant Decrease |
| | Nonmetropolitan | Segment 1 | 1999–2017 | 1.41 | (1.14, 1.67) | <0.001 | Significant Increase |
| | | Segment 2 | 2017–2020 | -2.95 | (-6.39, 0.61) | 0.097 | Stable |
| | | AAPC | 1999–2020 | 0.77 | (0.25, 1.30) | 0.004 | Significant Increase |
| State | Alabama | Segment 1 | 1999–2017 | 1.98 | (1.65, 2.30) | <0.001 | Significant Increase |
| | Alabama | Segment 2 | 2017–2024 | -3.98 | (-5.07, -2.89) | <0.001 | Significant Decrease |
| | | AAPC | 1999–2024 | 0.27 | (-0.10, 0.64) | 0.149 | Stable |
| | Alaska | Segment 1 | 1999–2024 | -1.70 | (-2.20, -1.19) | <0.001 | Significant Decrease |
| | | AAPC | 1999–2024 | -1.70 | (-2.20, -1.19) | <0.001 | Significant Decrease |
| | Arizona | Segment 1 | 1999–2016 | 0.52 | (0.16, 0.87) | 0.006 | Significant Increase |
| | | Segment 2 | 2016–2024 | -3.76 | (-4.65, -2.86) | <0.001 | Significant Decrease |
| | | AAPC | 1999–2024 | -0.87 | (-1.23, -0.52) | <0.001 | Significant Decrease |
| | Arkansas | Segment 1 | 1999–2017 | 3.12 | (2.70, 3.53) | <0.001 | Significant Increase |
| | | Segment 2 | 2017–2024 | -1.97 | (-3.30, -0.63) | 0.006 | Significant Decrease |
| | | AAPC | 1999–2024 | 1.67 | (1.21, 2.13) | <0.001 | Significant Increase |
| | California | Segment 1 | 1999–2017 | -1.34 | (-1.59, -1.08) | <0.001 | Significant Decrease |
| | | Segment 2 | 2017–2024 | -4.49 | (-5.49, -3.47) | <0.001 | Significant Decrease |
| | | AAPC | 1999–2024 | -2.23 | (-2.55, -1.91) | <0.001 | Significant Decrease |

| | | | | | | |
|---|---|---|---|---|---|---|
| Colorado | Segment 1 | 1999–2016 | -0.44 | (-0.68, -0.19) | 0.002 | Significant Decrease |
| | Segment 2 | 2016–2024 | -4.10 | (-4.78, -3.42) | <0.001 | Significant Decrease |
| | AAPC | 1999–2024 | -1.62 | (-1.88, -1.36) | <0.001 | Significant Decrease |
| Connecticut | Segment 1 | 1999–2017 | -1.00 | (-1.39, -0.61) | <0.001 | Significant Decrease |
| | Segment 2 | 2017–2024 | -3.99 | (-5.56, -2.38) | <0.001 | Significant Decrease |
| | AAPC | 1999–2024 | -1.84 | (-2.34, -1.34) | <0.001 | Significant Decrease |
| Delaware | Segment 1 | 1999–2016 | 1.02 | (0.23, 1.82) | 0.013 | Significant Increase |
| | Segment 2 | 2016–2024 | -5.32 | (-7.31, -3.28) | <0.001 | Significant Decrease |
| | AAPC | 1999–2024 | -1.05 | (-1.85, -0.24) | 0.011 | Significant Decrease |
| District of Columbia | Segment 1 | 1999–2002 | -9.13 | (-18.83, 1.73) | 0.092 | Stable |
| | Segment 2 | 2002–2011 | 2.91 | (0.34, 5.55) | 0.029 | Significant Increase |
| | Segment 3 | 2011–2024 | -3.51 | (-4.70, -2.32) | <0.001 | Significant Decrease |
| | AAPC | 1999–2024 | -1.96 | (-3.55, -0.34) | 0.018 | Significant Decrease |
| Florida | Segment 1 | 1999–2017 | 0.45 | (0.20, 0.69) | 0.001 | Significant Increase |
| | Segment 2 | 2017–2024 | -4.43 | (-5.30, -3.54) | <0.001 | Significant Decrease |
| | AAPC | 1999–2024 | -0.94 | (-1.23, -0.65) | <0.001 | Significant Decrease |
| Georgia | Segment 1 | 1999–2017 | 0.77 | (0.54, 0.99) | <0.001 | Significant Increase |
| | Segment 2 | 2017–2024 | -3.52 | (-4.27, -2.76) | <0.001 | Significant Decrease |
| | AAPC | 1999–2024 | -0.45 | (-0.70, -0.19) | <0.001 | Significant Decrease |
| Hawaii | Segment 1 | 1999–2013 | -1.61 | (-2.31, -0.89) | <0.001 | Significant Decrease |
| | Segment 2 | 2013–2017 | 4.01 | (-3.09, 11.63) | 0.258 | Stable |
| | Segment 3 | 2017–2024 | -3.26 | (-4.95, -1.54) | <0.001 | Significant Decrease |
| | AAPC | 1999–2024 | -1.20 | (-2.39, 0.01) | 0.051 | Stable |
| Idaho | Segment 1 | 1999–2019 | 0.30 | (-0.15, 0.75) | 0.182 | Stable |
| | Segment 2 | 2019–2024 | -5.35 | (-8.37, -2.23) | 0.002 | Significant Decrease |

| | | | | | | |
|---|---|---|---|---|---|---|
| | AAPC | 1999–2024 | -0.86 | (-1.55, -0.16) | 0.016 | Significant Decrease |
| Illinois | Segment 1 | 1999–2016 | 0.71 | (0.34, 1.08) | <0.001 | Significant Increase |
| | Segment 2 | 2016–2024 | -3.85 | (-4.91, -2.79) | <0.001 | Significant Decrease |
| | AAPC | 1999–2024 | -0.77 | (-1.18, -0.37) | <0.001 | Significant Decrease |
| Indiana | Segment 1 | 1999–2013 | 1.89 | (1.30, 2.49) | <0.001 | Significant Increase |
| | Segment 2 | 2013–2024 | -0.94 | (-1.67, -0.21) | 0.014 | Significant Decrease |
| | AAPC | 1999–2024 | 0.64 | (0.20, 1.07) | 0.004 | Significant Increase |
| Iowa | Segment 1 | 1999–2015 | 1.20 | (0.70, 1.69) | <0.001 | Significant Increase |
| | Segment 2 | 2015–2024 | -3.42 | (-4.49, -2.34) | <0.001 | Significant Decrease |
| | AAPC | 1999–2024 | -0.49 | (-0.97, -0.01) | 0.044 | Significant Decrease |
| Kansas | Segment 1 | 1999–2017 | 0.84 | (0.43, 1.25) | <0.001 | Significant Increase |
| | Segment 2 | 2017–2024 | -3.87 | (-5.38, -2.33) | <0.001 | Significant Decrease |
| | AAPC | 1999–2024 | -0.50 | (-1.00, -0.00) | 0.049 | Significant Decrease |
| Kentucky | Segment 1 | 1999–2015 | 1.79 | (1.31, 2.28) | <0.001 | Significant Increase |
| | Segment 2 | 2015–2024 | -1.75 | (-2.70, -0.80) | 0.001 | Significant Decrease |
| | AAPC | 1999–2024 | 0.50 | (0.06, 0.94) | 0.025 | Significant Increase |
| Louisiana | Segment 1 | 1999–2014 | 2.19 | (1.61, 2.77) | <0.001 | Significant Increase |
| | Segment 2 | 2014–2024 | -2.11 | (-3.01, -1.20) | <0.001 | Significant Decrease |
| | AAPC | 1999–2024 | 0.45 | (-0.03, 0.93) | 0.067 | Stable |
| Maine | Segment 1 | 1999–2017 | 0.11 | (-0.35, 0.58) | 0.615 | Stable |
| | Segment 2 | 2017–2024 | -3.49 | (-5.24, -1.72) | <0.001 | Significant Decrease |
| | AAPC | 1999–2024 | -0.91 | (-1.48, -0.34) | 0.002 | Significant Decrease |
| Maryland | Segment 1 | 1999–2006 | -1.93 | (-2.89, -0.95) | 0.001 | Significant Decrease |
| | Segment 2 | 2006–2009 | 2.68 | (-4.47, 10.37) | 0.440 | Stable |
| | Segment 3 | 2009–2014 | -3.43 | (-5.54, -1.27) | 0.005 | Significant Decrease |
| | Segment 4 | 2014–2018 | 1.08 | (-2.34, 4.62) | 0.508 | Stable |

| | | | | | | |
|---|---|---|---|---|---|---|
| | Segment 5 | 2018–2024 | -6.03 | (-7.14, -4.91) | <0.001 | Significant Decrease |
| | AAPC | 1999–2024 | -2.22 | (-3.26, -1.17) | <0.001 | Significant Decrease |
| Massachusetts | Segment 1 | 1999–2007 | -2.18 | (-3.15, -1.20) | <0.001 | Significant Decrease |
| | Segment 2 | 2007–2017 | 0.10 | (-0.72, 0.94) | 0.796 | Stable |
| | Segment 3 | 2017–2024 | -4.19 | (-5.34, -3.03) | <0.001 | Significant Decrease |
| | AAPC | 1999–2024 | -1.85 | (-2.37, -1.32) | <0.001 | Significant Decrease |
| Michigan | Segment 1 | 1999–2016 | 1.38 | (1.03, 1.73) | <0.001 | Significant Increase |
| | Segment 2 | 2016–2024 | -3.03 | (-3.97, -2.07) | <0.001 | Significant Decrease |
| | AAPC | 1999–2024 | -0.05 | (-0.42, 0.32) | 0.779 | Stable |
| Minnesota | Segment 1 | 1999–2017 | -0.07 | (-0.47, 0.33) | 0.726 | Stable |
| | Segment 2 | 2017–2024 | -4.60 | (-6.12, -3.06) | <0.001 | Significant Decrease |
| | AAPC | 1999–2024 | -1.36 | (-1.85, -0.86) | <0.001 | Significant Decrease |
| Mississippi | Segment 1 | 1999–2017 | 1.97 | (1.59, 2.35) | <0.001 | Significant Increase |
| | Segment 2 | 2017–2024 | -0.82 | (-2.10, 0.48) | 0.205 | Stable |
| | AAPC | 1999–2024 | 1.18 | (0.75, 1.62) | <0.001 | Significant Increase |
| Missouri | Segment 1 | 1999–2014 | 1.55 | (0.92, 2.19) | <0.001 | Significant Increase |
| | Segment 2 | 2014–2024 | -2.11 | (-3.12, -1.09) | <0.001 | Significant Decrease |
| | AAPC | 1999–2024 | 0.07 | (-0.46, 0.60) | 0.794 | Stable |
| Montana | Segment 1 | 1999–2017 | -0.34 | (-0.95, 0.27) | 0.259 | Stable |
| | Segment 2 | 2017–2024 | -3.71 | (-5.94, -1.43) | 0.003 | Significant Decrease |
| | AAPC | 1999–2024 | -1.30 | (-2.03, -0.56) | <0.001 | Significant Decrease |
| Nebraska | Segment 1 | 1999–2017 | 1.12 | (0.66, 1.58) | <0.001 | Significant Increase |
| | Segment 2 | 2017–2022 | -5.99 | (-9.95, -1.85) | 0.007 | Significant Decrease |
| | Segment 3 | 2022–2024 | 5.70 | (-7.71, 21.05) | 0.402 | Stable |
| | AAPC | 1999–2024 | 0.01 | (-1.31, 1.35) | 0.986 | Stable |
| Nevada | Segment 1 | 1999–2010 | -2.08 | (-3.55, -0.60) | 0.009 | Significant Decrease |

| | | | | | | |
|---|---|---|---|---|---|---|
| | Segment 2 | 2010–2015 | 1.70 | (-4.41, 8.21) | 0.574 | Stable |
| | Segment 3 | 2015–2024 | -3.90 | (-5.45, -2.32) | <0.001 | Significant Decrease |
| | AAPC | 1999–2024 | -2.00 | (-3.38, -0.60) | 0.005 | Significant Decrease |
| New Hampshire | Segment 1 | 1999–2024 | -0.98 | (-1.30, -0.65) | <0.001 | Significant Decrease |
| | AAPC | 1999–2024 | -0.98 | (-1.30, -0.65) | <0.001 | Significant Decrease |
| New Jersey | Segment 1 | 1999–2017 | 0.13 | (-0.33, 0.59) | 0.566 | Stable |
| | Segment 2 | 2017–2024 | -6.14 | (-7.92, -4.32) | <0.001 | Significant Decrease |
| | AAPC | 1999–2024 | -1.67 | (-2.25, -1.08) | <0.001 | Significant Decrease |
| New Mexico | Segment 1 | 1999–2017 | -0.13 | (-0.63, 0.36) | 0.577 | Stable |
| | Segment 2 | 2017–2024 | -3.29 | (-5.06, -1.49) | 0.001 | Significant Decrease |
| | AAPC | 1999–2024 | -1.03 | (-1.61, -0.44) | <0.001 | Significant Decrease |
| New York | Segment 1 | 1999–2018 | -0.69 | (-0.93, -0.45) | <0.001 | Significant Decrease |
| | Segment 2 | 2018–2024 | -5.80 | (-7.14, -4.44) | <0.001 | Significant Decrease |
| | AAPC | 1999–2024 | -1.94 | (-2.30, -1.58) | <0.001 | Significant Decrease |
| North Carolina | Segment 1 | 1999–2016 | 0.59 | (0.26, 0.93) | 0.001 | Significant Increase |
| | Segment 2 | 2016–2024 | -3.72 | (-4.60, -2.83) | <0.001 | Significant Decrease |
| | AAPC | 1999–2024 | -0.81 | (-1.15, -0.46) | <0.001 | Significant Decrease |
| North Dakota | Segment 1 | 1999–2015 | 0.82 | (-0.07, 1.72) | 0.070 | Stable |
| | Segment 2 | 2015–2024 | -3.12 | (-5.09, -1.11) | 0.004 | Significant Decrease |
| | AAPC | 1999–2024 | -0.62 | (-1.49, 0.26) | 0.166 | Stable |
| Ohio | Segment 1 | 1999–2016 | 0.68 | (0.34, 1.03) | <0.001 | Significant Increase |
| | Segment 2 | 2016–2024 | -2.89 | (-3.86, -1.90) | <0.001 | Significant Decrease |
| | AAPC | 1999–2024 | -0.47 | (-0.85, -0.10) | 0.013 | Significant Decrease |
| Oklahoma | Segment 1 | 1999–2009 | 3.59 | (2.51, 4.69) | <0.001 | Significant Increase |
| | Segment 2 | 2009–2024 | -0.44 | (-0.92, 0.04) | 0.071 | Stable |
| | AAPC | 1999–2024 | 1.15 | (0.67, 1.64) | <0.001 | Significant Increase |

| | | | | | | |
|---|---|---|---|---|---|---|
| Oregon | Segment 1 | 1999–2015 | -0.26 | (-0.65, 0.12) | 0.171 | Stable |
| | Segment 2 | 2015–2024 | -3.36 | (-4.19, -2.52) | <0.001 | Significant Decrease |
| | AAPC | 1999–2024 | -1.39 | (-1.76, -1.02) | <0.001 | Significant Decrease |
| Pennsylvania | Segment 1 | 1999–2015 | 0.49 | (0.08, 0.91) | 0.022 | Significant Increase |
| | Segment 2 | 2015–2024 | -3.21 | (-4.15, -2.27) | <0.001 | Significant Decrease |
| | AAPC | 1999–2024 | -0.86 | (-1.27, -0.45) | <0.001 | Significant Decrease |
| Rhode Island | Segment 1 | 1999–2015 | -0.35 | (-1.09, 0.39) | 0.332 | Stable |
| | Segment 2 | 2015–2024 | -3.79 | (-5.55, -2.01) | <0.001 | Significant Decrease |
| | AAPC | 1999–2024 | -1.61 | (-2.36, -0.85) | <0.001 | Significant Decrease |
| South Carolina | Segment 1 | 1999–2017 | 1.24 | (0.91, 1.58) | <0.001 | Significant Increase |
| | Segment 2 | 2017–2024 | -4.65 | (-5.77, -3.51) | <0.001 | Significant Decrease |
| | AAPC | 1999–2024 | -0.44 | (-0.83, -0.06) | 0.024 | Significant Decrease |
| South Dakota | Segment 1 | 1999–2009 | 2.80 | (0.92, 4.72) | 0.005 | Significant Increase |
| | Segment 2 | 2009–2024 | -1.57 | (-2.48, -0.66) | 0.002 | Significant Decrease |
| | AAPC | 1999–2024 | 0.16 | (-0.71, 1.03) | 0.727 | Stable |
| Tennessee | Segment 1 | 1999–2017 | 1.30 | (0.98, 1.62) | <0.001 | Significant Increase |
| | Segment 2 | 2017–2024 | -2.47 | (-3.55, -1.38) | <0.001 | Significant Decrease |
| | AAPC | 1999–2024 | 0.23 | (-0.13, 0.59) | 0.217 | Stable |
| Texas | Segment 1 | 1999–2017 | 0.35 | (0.06, 0.63) | 0.019 | Significant Increase |
| | Segment 2 | 2017–2024 | -4.18 | (-5.18, -3.17) | <0.001 | Significant Decrease |
| | AAPC | 1999–2024 | -0.94 | (-1.27, -0.61) | <0.001 | Significant Decrease |
| Utah | Segment 1 | 1999–2012 | -0.49 | (-1.22, 0.24) | 0.176 | Stable |
| | Segment 2 | 2012–2016 | 3.88 | (-2.12, 10.24) | 0.195 | Stable |
| | Segment 3 | 2016–2024 | -2.35 | (-3.52, -1.16) | <0.001 | Significant Decrease |
| | AAPC | 1999–2024 | -0.41 | (-1.42, 0.61) | 0.433 | Stable |
| Vermont | Segment 1 | 1999–2011 | -0.50 | (-1.69, 0.70) | 0.392 | Stable |

| | | | | | | |
|---|---|---|---|---|---|---|
| | Segment 2 | 2011–2024 | -3.50 | (-4.54, -2.46) | <0.001 | Significant Decrease |
| | AAPC | 1999–2024 | -2.08 | (-2.82, -1.33) | <0.001 | Significant Decrease |
| Virginia | Segment 1 | 1999–2018 | -0.59 | (-0.92, -0.25) | 0.001 | Significant Decrease |
| | Segment 2 | 2018–2024 | -3.08 | (-4.73, -1.40) | 0.001 | Significant Decrease |
| | AAPC | 1999–2024 | -1.19 | (-1.64, -0.74) | <0.001 | Significant Decrease |
| Washington | Segment 1 | 1999–2015 | -1.05 | (-1.51, -0.59) | <0.001 | Significant Decrease |
| | Segment 2 | 2015–2024 | -4.53 | (-5.56, -3.49) | <0.001 | Significant Decrease |
| | AAPC | 1999–2024 | -2.32 | (-2.77, -1.86) | <0.001 | Significant Decrease |
| West Virginia | Segment 1 | 1999–2017 | 1.11 | (0.65, 1.57) | <0.001 | Significant Increase |
| | Segment 2 | 2017–2024 | -2.29 | (-3.98, -0.57) | 0.012 | Significant Decrease |
| | AAPC | 1999–2024 | 0.15 | (-0.40, 0.70) | 0.600 | Stable |
| Wisconsin | Segment 1 | 1999–2017 | 0.34 | (0.08, 0.60) | 0.013 | Significant Increase |
| | Segment 2 | 2017–2024 | -3.52 | (-4.50, -2.52) | <0.001 | Significant Decrease |
| | AAPC | 1999–2024 | -0.75 | (-1.07, -0.43) | <0.001 | Significant Decrease |
| Wyoming | Segment 1 | 1999–2024 | -1.01 | (-1.42, -0.60) | <0.001 | Significant Decrease |
| | AAPC | 1999–2024 | -1.01 | (-1.42, -0.60) | <0.001 | Significant Decrease |

APC, Annual Percent Change; AAPC, Average Annual Percent Change; CI, Confidence Interval.

Significant trend directions (Significant Increase/Significant Decrease) are defined based on statistical significance ($P < 0.05$).

Stable indicates that the APC or AAPC is not statistically significantly different from zero ($P >= 0.05$).

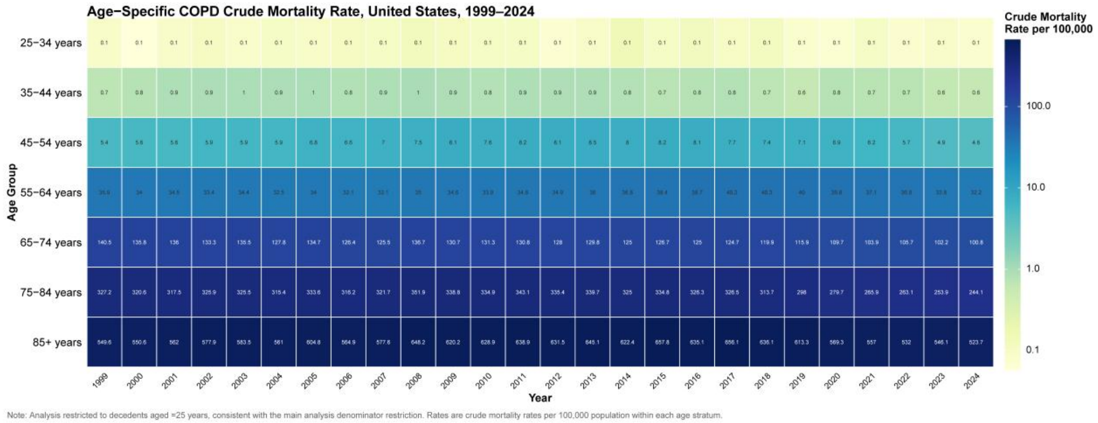


**Supplementary Figure S1.Heatmap of age-specific COPD mortality rates in the United States, 1999–2024.**

Cells represent crude COPD mortality rates per 100,000 population by year and age group, illustrating the steep age gradient and sustained concentration of burden in older adults.

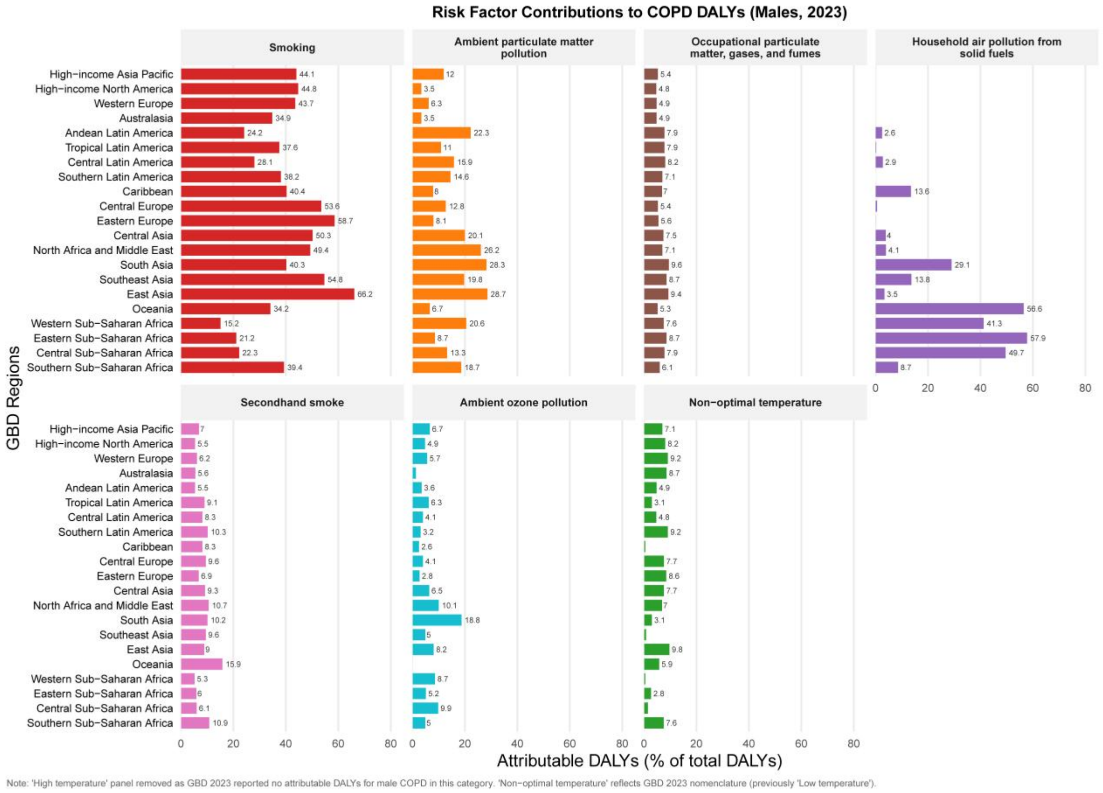


**Supplementary Figure S2.**Regional contributions of major risk factors to male COPD disability-adjusted life years (DALYs), GBD 2023. The figure shows the proportion of total COPD DALYs attributable to smoking, ambient particulate matter pollution, occupational particulate matter/gases/fumes, household air pollution from solid fuels, secondhand smoke, ambient ozone pollution, and non-optimal temperature exposure across GBD super-regions. Data were obtained from the GBD 2023 Results Tool (Institute for Health Metrics and Evaluation). This figure pertains to males only.
